# Differences in central facilitation between episodic and chronic migraineurs in nociceptive-specific trigeminal pathways

**DOI:** 10.1186/s10194-016-0637-6

**Published:** 2016-04-16

**Authors:** Jong-Hee Sohn, Chul-Ho Kim, Hui-Chul Choi

**Affiliations:** Department of Neurology, Chuncheon Sacred Heart Hospital, Hallym University College of Medicine, 153 Gyo-dong, Chuncheon-si, Gangwon-do 200-704 Republic of Korea

**Keywords:** Chronic migraine, Episodic migraine, Nociceptive blink reflex, Pain-related evoked potential, Trigeminal pathway

## Abstract

**Background:**

The trigeminal nociceptive system plays a pivotal role in the pathophysiology of migraines. The present study investigated whether there are differences between patients with episodic migraine (EM) and patients with chronic migraine (CM) in trigeminal pain processing at the brainstem and cortical levels using the nociceptive blink reflex (nBR) and pain-related evoked potentials (PREP).

**Methods:**

This study assessed 68 female migraineurs (38 EM patients and 30 CM patients) and 40 age-matched controls using simultaneous recordings of nBR and PREP during the interictal period.

**Results:**

In terms of the nBR, EM patients displayed significantly decreased latencies and larger amplitudes and area-under-the-curve (AUC) values for the R2 component, whereas CM patients showed significantly prolonged latencies and smaller amplitudes and AUC values for the R2 component (*p* < 0.05). In terms of PREP, both the EM and CM patients had decreased latencies (N1, P1), with larger amplitude compared with the controls (*p* < 0.05), which indicates facilitation at the cortical level. Additionally, the amplitude and AUC values of the R2 component exhibited a negative correlation, whereas the latency of the R2 component for the nBR showed a positive correlation, with the frequency of headaches in migraineurs (*p* < 0.01).

**Conclusions:**

In the present study, the facilitation in the trigeminal nociceptive pathway of the EM group suggests the occurrence of migraine-specific hyperexcitability. Additionally, the suppression of R2 at the brainstem level in the CM group may relate to impaired or dysfunctional descending pain modulation. These findings suggest that there are adaptive or maladaptive responses due to the chronification of migraine attacks.

## Background

Chronic migraine (CM) is a disabling neurological condition, accepted by the main body of the current beta version of the International Classification of Headache Disorders-3 (ICHD-3β) [1]. This classification indicates that the chronicity of the disorder is not a complication of migraine, but rather a transformation from an episodic to a chronic disorder. Approximately 3 % of individuals with episodic migraine (EM) progress to CM over the course of a year [2]. Despite the spectrum conceptualization of migraine, there are important distinctions between EM and CM. Several studies have provided data on differences in symptoms, comorbidity profiles, disabilities, and treatment responses of CM versus EM [3–6]. Moreover, differences in pathophysiology and functional correlates observed in electrophysiological and imaging studies have been noted [7]. Although the precise mechanisms underlying headache chronification from EM to CM are not fully understood, the central sensitization and dysfunctional pain control systems are thought to be involved [8].

The trigeminal nociceptive system plays a pivotal role in the pathophysiology of migraine. The use of neurophysiological methods to explore pain-related circuits is an important aid in headache research. To increase nociceptive sensitivity, previous studies have developed a novel technique of selective electrical transcutaneous nociceptive fiber stimulation using a custom concentric planar electrode. The concentric electrode allows quantitative measurements of trigeminal nociception transmission and is highly sensitive to changes in trigeminal nociception [9–13]. Investigations of trigeminal nociceptive pathways during ictal phases have been recorded in EM, and these data suggest temporary and specific sensitization of central trigeminal neurons during acute migraine attacks [9, 14]. In addition, the possibility that increased excitability of the trigeminal pathways may persist during the interictal period has been proposed in EM [15]; however, investigations of trigeminal sensory pathways in CM, particularly in regard to nociceptive processing, are rare. One previous study did not observe any changes [16], whereas other studies observed abnormal excitability [13, 17]. These inconsistencies may result from the use of different study techniques, such as the conventional blink reflex, or different study groups, such as in individuals with combined medication-overuse headaches. It has been assumed that these abnormalities would be more marked in CM, and that there are distinctions between EM and CM.

Thus, we hypothesized that the nociceptive trigeminal pathway may be altered to varying degrees in patients with EM and CM. We assessed trigeminal nociceptive processing in patients with EM and CM during the interictal period compared to healthy controls to investigate the presence or absence of facilitation processes in the patient group. To determine whether there were differences in trigeminal pain processing at the brainstem and cortical levels, we used the nociceptive blink reflex (nBR) and pain-related evoked potentials (PREP) in EM and CM patients.

## Methods

### Subjects

This study collected data from patients with EM and CM treated in the headache clinic of a university hospital between October 2014 and June 2015. All participants were between 20 and 60 years of age, and only females were included to eliminate age and gender bias. They were examined and classified according to the ICHD-3β by a board-certified neurologist based on patient history, a neurologic examination, and neuroimaging study. Additionally, patients were required to have at least a 1-year migraine history prior to enrollment, to exclude other primary headaches. 30 patients with CM and 38 patients with EM were investigated in a case–control design. The control group consisted of 40 age-matched female volunteers. We recruited the control group via advertisements such as posted notices in the hospital. Control participants had to be devoid of headaches for at least 3 months prior to enrollment, have no personal or family history of migraine, and have no more than an occasional mild headache (<5 times per year); we also included only those participants who had not sought medical treatment for headaches. Exclusion criteria included subjects with prophylactic daily medication to prevent headaches, concomitant medication-overuse headache based on ICHD-3β, neurological disorders, any other systemic disease, history of Bell’s palsy, somatic or psychiatric illness (e.g., depression, anxiety disorder), use of oral contraceptives, and pregnancy.

The patients with migraine were asked to complete a headache questionnaire during the last one month. Headache frequency (days/month) by the number of days with headaches for a month, duration (hours/day) by the sum of the total hours of headaches per attack, and intensity (numeric rating scale [NRS]: 0 = no pain to 10 = unbearable pain) by calculating the mean of the NRS for the days with headaches were described. All participants were instructed to refrain from caffeine, nicotine, and alcohol for at least 24 h prior to testing. This study protocol and informed consent form were reviewed and approved by the local Institutional Review Board (Institutional Review Board/Ethics Committee of Hallym University Chuncheon Sacred Heart Hospital). Written informed consent was obtained from all participants prior to enrollment in the study.

### Experimental procedure

The recording sessions were conducted at the same time of day (2–5 PM) by expert neurophysiologists (J-H, J) blind to the clinical diagnosis of the subjects. Recordings were performed using a Nicolet EDX EMP/EP machine (Natus Neurology, Middleton, WI, USA). Patients with migraine underwent testing during headache-free days. Specifically, recordings in EM patients were obtained interictally at least 2 days after the last and before the next migraine attack. All CM patients underwent the recordings in the interictal period (at least 2 days before and after a typical migraine attack), but a current background mild headache (NRS < 3) was allowed. Following the test, patients were contacted by telephone and excluded from the study if they had experienced a migraine attack within 2 days of the recording. The participants were in a lying position with their eyes closed during the recordings. Two planar concentric surface stimulating electrodes were used for nociceptive stimulation (inomed Medizintechnik GmbH, Emmendingen, Germany, http://www.inomed.com).

Individual sensory (Is) and pain (Ip) perception thresholds were defined as the minimum stimulation intensity perceived as tactile and painful, respectively. We first determined sensory and pain threshold on both sides of the forehead using ascending and descending single pulses in 0.1 mA steps, with a duration of 0.5 ms and an interstimulus interval of 15–20 s (randomized interval). For patients’ comfort and to avoid a lengthy procedure, we performed unilateral stimulation for the nBR and PREP recordings. The concentric electrode was placed on the left lower forehead, approximately 10 mm above the supraorbital foramen, to stimulate the supraorbital nerve. nBR and PREP were recorded simultaneously following trigeminal stimulation of the forehead A fixed stimulation intensity (SI) of 1.5 × Ip was used to evoke the nBR and PREP. Trains of electrical stimulation composed of three pulses (each 0.5 ms in duration) with a 5 ms interpulse interval were applied to further increase nociceptive specificity [12], and stimulation intensities > 2 mA were excluded to minimize the risk of A*β* fiber co-activation [10]. Repeated stimulation of the train pulse was delivered at random intervals of 18–22 s to obtain at least 11 consecutive responses. Surface recording electrodes for nBR were placed on the infraorbital area (active) and at the base of the nose with a 2 Hz to 1 kHz band-pass filter (sampling rate 2.5 kHz, 150 ms sweep length, 200 ms analysis time, and 100 μV sensitivity), which was used for recording. PREP was recorded with electrodes placed at Cz linked to both earlobes (A1–A2) based on the international 10–20 system.

### Analysis

An investigator who was blind to the diagnosis performed signal analyses. All recordings were averaged offline using the Synergy Reader software, version 20.1 (Natus Neurology). The first recording was excluded from signal analysis to avoid contamination with startle response.

For the nBR recording, the 10 responses were rectified and averaged offline. The onset latency was visually determined as the initial point from the baseline (ms), and the AUC was determined between 27 ms and 87 ms of the R2 component (mV*ms) [18]. The following parameters were measured: the onset latency (ms), the mean amplitude of the root mean square (RMS, μV), and the area under the curve (AUC, mV*ms) values from the ipsilateral and contralateral R2 (iR2 and cR2).

For the PREP recording, the 10 responses were averaged offline. The negative (N1) and positive (P1) peak latencies and peak-to-peak amplitudes (PPA) were analyzed. The following parameters were measured: the peak latency of N1 and P1 (ms) and the mean of the PPA (μV).

### Statistics

Statistical analyses were performed using SPSS software ver. 21.0 for Windows (SPSS, Inc., Chicago, IL, USA). All values are reported as means and standard deviations. The Kruskal-Wallis test was used to compare the thresholds and parameters of nBR and PREP between groups. The Mann–Whitney *U* test for multiple comparisons was performed for the *post-hoc* analyses. Spearman’s correlation analysis was used to explore the relationship between electrophysiologic parameters and headache parameters (i.e., frequency, duration, and intensity). Results were considered significant at a *p*-value < 0.05.

## Results

### Clinical characteristics

No significant differences in age were noted (EM: 40.95 years, CM: 43.07 years, controls: 43.73 years). The frequency of headaches and the duration of the headaches according to the headache questionnaire were 4.81 days/month and 14.71 h/day, in patients with EM and 20.03 days/month and 16.00 h/day, respectively, in patients with CM. The mean intensity per episode (NRS) was 7.74 in patients with EM and 7.96 in patients with CM. The duration of having the headache condition was 9.93 years and 14.13 in patients with EM and CM, respectively (Table 1).

**Table 1:** Demographic data and headache features

	EM	CM	Control	*P*-value
	*n* = 38	*n* = 30	*n* = 40	
Age (years)	40.95 ± 10.00	43.07 ± 11.09	43.73 ± 11.75	NS
Frequency (days/month)	4.81 ± 3.30	20.03 ± 4.39	-	0.00
Duration (hours/day)	14.71 ± 8.44	16.00 ± 8.46	-	NS
Intensity (NRS: 0–10)	7.74 ± 1.91	7.96 ± 1.67	-	NS
Disease duration (years)	9.93 ± 9.06	14.13 ± 10.00	-	0.07

Values are expressed as mean ± standard deviation

*CM* chronic migraine, *EM* episodic migraine, *NS* not significant, *NRS* numeric rating scale

### Sensory and pain thresholds

The mean values for sensory and pain perception thresholds (mA) were as follows: 0.20 ± 0.09, 0.78 ± 0.30 in EM, 0.19 ± 0.05, 0.77 ± 0.26 in CM, and 0.20 ± 0.10, 0.84 ± 0.35 in controls. Patients with CM displayed lower sensory and pain perception thresholds during the recording; however, no significant differences were noted between subject groups. The mean values for pain stimulation intensity of each group was 1.17 (confidence interval, CI, 0.32–1.98) in EM, 1.16 (CI, 0.59–1.95) in CM, and 1.27 (CI, 0.32–1.98) in controls.

### Nociceptive blink reflex

**Fig. 1:**
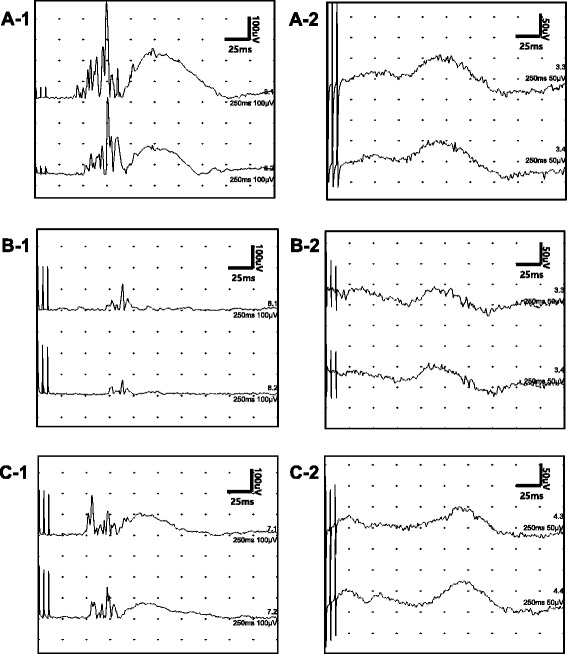
Examples of the nBR and PREP in patients with episodic and chronic migraine and controls. **A-1** nBR in an EM patient, **A-2** PREP in an EM patient, **B-1** nBR in a CM patient, **B-2** PREP in a CM patient, **C-1** nBR in a control patient, **C-2** PREP in a control patient. nBR, nociceptive blink reflex; PREP, pain-related evoked potential; EM, episodic migraine; CM, chronic migraine

The nBR results related to trigeminal nociceptive processing at the brainstem level are summarized in Table 2. Examples of an nBR recording from a patient with EM, a patient with CM, and a healthy control are shown in Fig. 1. The mean amplitude (RMS) and AUC values from the ipsilateral and contralateral R2 components (iR2 and cR2) were significantly lower in CM patients than in control and EM patients. Additionally, significantly prolonged latencies of the R2 component were seen in patients with CM (iR2 in CM vs. controls, iR2 and cR2 in CM vs. EM, *p* < 0.05). However, EM patients displayed significantly decreased latencies and larger amplitudes and AUC values of both R2 components in nBR (*p* < 0.05).

**Table 2:** Nociceptive blink reflex parameters

	iR2 of nBR			cR2 of nBR		
	Latency (ms)	Amplitude (μ)	AUC (mV *ms)	Latency (ms)	Amplitude (μ)	AUC (mV *ms)
EM	42.76 ± 3.90**^,^ ***	306.50 ± 126.17**^,^ ***	3.50 ± 1.87**^,^ ***	44.96 ± 3.69**^,^ ***	238.55 ± 85.95**^,^ ***	2.84 ± 1.28**^,^ ***
CM	48.44 ± 5.04*^,^ ***	154.56 ± 41.66*^,^ ***	1.59 ± 0.54*^,^ ***	51.02 ± 5.89***	111.78 ± 43.73*^,^ ***	1.11 ± 0.37*^,^ ***
Controls	45.47 ± 3.92	198.68 ± 75.01	2.24 ± 1.00	48.18 ± 4.16	177.81 ± 86.04	2.16 ± 1.30

*p < 0.05 between CM and controls; **p < 0.05 between EM and controls; ***p < 0.05 between CM and EM. *P*-values were determined using the Mann–Whitney *U* test

All values are expressed as means and standard deviations. *EM* episodic migraine, *CM* chronic migraine, *nBR* nociceptive blink reflex, *iR2* ipsilateral R2 component, *cR2* contralateral R2 component, *AUC* area under the curve

### Pain-related evoked potentials

The PREP results regarding trigeminal nociceptive processing at the cortical level are summarized in Table 3. Examples of a PREP recording from patients with EM and CM and a healthy control are shown in Fig. 1. EM patients displayed significantly decreased latencies (both N1 and P1), with larger right amplitude (PPA), compared with controls (*p* < 0.05). Additionally, CM patients showed significantly decreased latencies (left N1, left and right P1), with larger right amplitude (PPA), compared with controls (*p* < 0.05). However, no significant differences were found in the PREP parameters between patients with EM and CM.

**Table 3:** Pain-related evoked potential parameters

	Left PREP			Right PREP		
	N1 latency (ms)	P1 latency (ms)	PPA (μ)	N1 latency (ms)	P1 latency (ms)	PPA (μ)
EM	122.62 ± 9.71**	169.82 ± 9.70**	63.76 ± 13.22	121.37 ± 10.09**	169.56 ± 10.61**	63.75 ± 13.35**
CM	125.25 ± 8.71*	172.83 ± 10.43*	61.05 ± 15.65	125.95 ± 8.46	172.47 ± 10.43*	59.13 ± 15.74*
Controls	130.71 ± 6.84	179.10 ± 8.50	59.48 ± 14.25	130.14 ± 6.94	178.59 ± 8.05**	54.63 ± 13.91

*p < 0.05 between CM and controls; **p < 0.05 between EM and controls; there are no statistical differences between CM and EM for PREP parameters. *P*-values were determined using the Mann–Whitney *U* test. All values are expressed as means and standard deviations

*EM* episodic migraine, *CM* chronic migraine, *PREP* pain-related evoked potential, *PPA* peak-to-peak amplitude, *AUC* area under the curve

### Correlations between headache parameters and electrophysiologic parameters

To explore possible clinical–electrophysiological correlations, we examined the correlations between headache parameters, including frequency, duration, and intensity, and nBR and PREP parameters. Although the latency of the R2 component in the nBR was positively correlated with the frequency of headache, the amplitude and AUC from the R2 component in the nBR were negatively correlated with the frequency of headache parameters in the migraine group (*p* < 0.01) (Table 4). However, apart from these, no significant correlations were noted between nBR and PREP parameters and other headache parameters.

**Table 4:** Correlation coefficients between headache characteristics and parameters of the nBR and the PREP in migraineurs

	Correlation coefficient (*r*) for the nBR parameters		
	Latency	Amplitude	AUC
Frequency of headache attacks (days)	0.497 (iR2)**	−0.588 (iR2)**	−0.521 (iR2)**
	0.469 (cR2)**	−0.634 (cR2)**	−0.626 (cR2)**
Duration of headache attacks (hours)	NS	NS	NS
Pain intensity of headache attacks (NRS)	NS	NS	NS

Spearman’s correlation test (*r*), **p < 0.01. *EM* episodic migraine, *CM* chronic migraine, *PREP* pain-related evoked potential, *NRS* numeric rating scale, *NS* non-significant, *nBR* nociceptive blink reflex, *iR2* ipsilateral R2 component, *cR2* contralateral R2 component, *AUC* area under the curve, *PREP* pain-related evoked potential, *PPA* peak-to-peak amplitude

## Discussion

Our results showed R2 suppression at the brainstem level (nBR) and facilitation at the cortical level (PREP) of trigeminal pain processing in CM patients, and facilitation at both the brainstem and cortical levels in EM patients. In addition, for the nBR, a significant correlation between headache frequency and R2 suppression (prolonged latency, low amplitude, low AUC) was demonstrated in migraineurs.

A variety of nociceptive stimuli are able to elicit PREP at the cortical level and can be used to assess spinothalamocortical pain-temperature pathways [19, 20]. Previous clinical studies have demonstrated the facilitation of the trigeminal nociceptive system in a pain-free state with event-related potentials following CO_2_ laser stimulation (LEP) in CM and chronic tension-type headache [21, 22]. In patients with medication overuse headache, activation of trigeminal and somatic PREP, but not of nBR was also found [13]. However, these central facilitation changes at the cortical level have not been observed in other chronic headache disorders, such as hypnic headache [23]. Thus, it is not clear whether this observed phenomenon is the cause or the consequence of the headache chronification.

In our study, facilitation of trigeminal PREP during the inter-ictal period was observed in both the EM and CM groups. The cingulate cortex has been thought to play a major role in cortical plasticity as the main generator of PREP or LEP [24, 25]. Patients with EM have shown deficient habituation with LEP represented by an abnormal excitability of cortical areas during interictal phase [26]. Several studies have shown modulation of the habituation deficit in LEP induced by preventive medication and by high-frequency repetitive transcranial magnetic stimulation [27, 28]. This deficient habituation in the LEP was also partly restored after treatment of medication overuse headache [29]. These experimental studies reflect a modification in pain-processing pathways, which may constitute an important dysfunction, that probably forms the background in migraineurs. Thus, the facilitation of trigeminal nociception at the cortical level may be specific to migraineurs rather than to the chronification of pain.

The R2 component of the blink reflex reflects the excitability of brainstem interneurons and the function of synaptic transmission in the brainstem [30]. Previous studies have reported that the R2 component of the nBR by nociceptive stimuli was more than six times greater during migraine attacks [9]; these facilitation changes are specific to episodic migraines and act as the central driving force for migraine attacks [14]. Several studies using neurophysiologic tools to explore the trigeminal system during the interictal period found that patients with EM have a lack of habituation of nBR and enhanced R2 recovery of BR and corneal reflex abnormality [15, 31, 32]. Our results showing R2 facilitation of nBR between attacks in EM patients are consistent with these findings. These phenomena suggest that sensitization of the trigeminal system may persist interictally. Another interesting finding is that asymptomatic individuals with a family history of migraine present the same nBR abnormalities as patients with full-brown migraine between attacks [33]. On the other hand, another study found normal the R2 recovery curves between attacks in EM patients and these results do not support persistent interictal sensitization in the spinal trigeminal sensory system [34].

However, in terms of the nBR, R2 suppression occurred in the CM group, which differed from the EM group in our study in this regard. Additionally, each parameter of the nBR (latency, amplitude, and AUC) was positively or negatively correlated with headache frequency in migraineurs. Thus, the degree of R2 suppression seems to be correlated with the number of headache days. Brainstem interneuronal excitability is assumed to be under the control of rostral structures, and the R2 component of the blink reflex is largely influenced by suprasegmental control, that comes mainly from the cerebral cortex and basal ganglia [35, 36]. The descending pain modulation system might be responsible for keeping the primary value of the nBR within certain physiological boundaries with respect to homeostatic neuronal plasticity [37]. These networks of cortical and subcortical structures with modulatory nociceptive and antinociceptive functions become abnormally activated during, or even between, migraine attacks [38]. During repeated migraine attacks, modification of this network can occur. Therefore, excessive R2 suppression at the brainstem level may be related to an impaired or dysfunctional descending pain modulation system. These findings may relate to adaptive or dysregulated/maladaptive mechanisms within the context of the allostatic load model [39].

Excitability changes in the blink reflex circuit can also occur as a consequence of damage in the neural structures along the blink reflex circuit [36]. Several neuroimaging studies on migraineurs reported morphological and functional abnormalities in the brainstem [40–43]. In particular, EM patients demonstrated activation of the rostral brainstem during the interictal phase in functional MRI and exhibited increased density in the periaqueductal gray matter in structural imaging using voxel-based morphometry [42, 43]. MRI findings of iron accumulation in the brainstem have been correlated with both duration of illness and frequency of attacks in migraineurs [44]. Additionally, CM patients showed persistent brainstem dysfunction in positron emission tomography evaluations and brainstem atrophy in high-resolution anatomical MRI images [45, 46]. A functional imaging study using ^1^H-magnetic resonance spectroscopy showed an increased N-acetyl aspartate/creatinine (NAA/Cr) ratio suggestive of neuronal hypertrophy at the dorsal pons in EM compared with controls. Headache frequency and intensity were negatively correlated with the NAA/Cr ratio. These findings suggest that after an initial response of hypertrophy in EM, there may be neuronal loss in CM in these regions [47]. Thus, these plastic structural changes according to migraine status may influence the R2 facilitation or suppression of the nBR in migraineurs. Repeated episodes of central sensitization may be associated with neuronal damage at the brainstem level, with resultant poor modulation of pain, and migraine progression [48]. However, it is currently unclear whether these structural brain changes seen in migraineurs are the cause or the result of headaches.

Our study had several limitations. Patients at a specialized headache clinic were recruited, and the size of the sample was small. Additionally, the study used a cross-sectional design that provided limited information. To further understand the differences between EM and CM, longitudinal electrophysiological follow-up studies are warranted. Moreover, we used only unilateral stimulation for recording to avoid an excessively lengthy procedure and to enhance patients’ comfort. We believe that these decisions are probably not relevant to the interpretation of our results, because none of our patients had fixed unilateral migraine attacks, and there were no side differences in stimulus detection and pain threshold measurements. In addition, only females were included in the present study because of potential effects of sex on the blink reflex [49]; therefore, we are unable to generalize to men. A larger study sample will be required for a better and improved understanding of the relationship between measured electrophysiological factors and clinical findings.

## Conclusions

In conclusion, this study provides electrophysiological evidence that excitability of nociceptive-specific trigeminal pathways is different between EM and CM. Facilitation of trigeminal nociceptive processing was observed in EM, whereas R2 suppression at the brainstem level and additional central facilitation changes at the cortical level were seen in CM. Facilitation along trigeminal nociceptive processing in EM patients was associated with migraine-specific hyperexcitability due to intrinsically increased excitability or impaired intra-inhibitory mechanisms. In CM, additional R2 suppression at the brainstem level may be related to impaired or dysfunctional descending pain modulation. These changes in CM are suggestive of adaptive or maladaptive responses due to repetitive episodes of migraine attacks.

## Abbreviations

AUC: area under the curve
CM: chronic migraine
cR2: contralateral R2 component
EM: episodic migraine
ICHD-3β: beta version of the International Classification of Headache Disorders-3
Ip: pain perception threshold
iR2: ipsilateral R2 component
Is: sensory perception threshold
N1: negative peak
nBR: nociceptive blink reflex
NRS: numeric rating scale
P1: positive peak
PPA: peak-to-peak amplitude
PREP: pain-related evoked potentials
RMS: root mean square
SI: stimulation intensity
